# Health literacy and psychological wellbeing of employees working from home in Germany—online survey results

**DOI:** 10.1093/heapro/daae202

**Published:** 2025-01-17

**Authors:** Lara Kleist, Lukas Kühn, Eileen Wengemuth, Kyung-Eun (Anna) Choi

**Affiliations:** Center for Health Services Research, Brandenburg Medical School Theodor Fontane, Fehrbelliner Straße 38, 16816 Neuruppin, Germany; Center for Health Services Research, Brandenburg Medical School Theodor Fontane, Fehrbelliner Straße 38, 16816 Neuruppin, Germany; Center for Health Services Research, Brandenburg Medical School Theodor Fontane, Fehrbelliner Straße 38, 16816 Neuruppin, Germany; Center for Health Services Research, Brandenburg Medical School Theodor Fontane, Fehrbelliner Straße 38, 16816 Neuruppin, Germany; Health Services Research, Research Center Medical Imaging and Artificial Intelligence (MIAAI), Danube Private University (DPU) GmbH, Steiner Landstraße 124, 3500 Krems-Stein, Austria

**Keywords:** mental health, organizational health literacy, work-related health literacy, home office, employee health

## Abstract

Employees’ psychological wellbeing is of special interest to employers, as mental illnesses are still the second most common reason for work absences. The psychological wellbeing of employees is determined by factors at an individual, interpersonal and organizational level. Health literacy encompasses both the individual and the organizational level and thus offers a good concept against the background of employees’ psychological wellbeing. Furthermore, demographic change increases skills shortages, while recently, other working models, such as increasing home office arrangements, benefit and challenge both employees and employers. Therefore, this study examines the associations between individual and organizational health literacy, work-from-home culture, and the psychological wellbeing of employees who mainly work from home. An anonymous open online survey via Facebook and Instagram advertisements was conducted in June 2023 in Germany. The questionnaire included nine thematic groups with validated and nonvalidated scales (e.g. WHO-5 Wellbeing-Index). Data from 103 participants were suitable for data analyses in IBM SPSS Statistics 23. Of the participating employees, 17% were assigned to males and 83% to females. The mean age was 49.5 years. Individual and organizational health literacy and work-from-home culture were positively associated with employees’ psychological wellbeing. Organizational health literacy mediated the effect of individual health literacy on employees’ psychological wellbeing. Individual and organizational health literacy totally mediated the impact of work-from-home culture. The study results highlight that individual and organizational health literacy provide useful concepts for practitioners and researchers regarding the psychological wellbeing of employees working from home and that both might play a crucial role in mediating the effect of organizational culture aspects on employees’ psychological wellbeing.

**Contribution to Health Promotion**

- Individual and organizational health literacy provide useful concepts for practitioners and researchers when it comes to the psychological wellbeing of employees who are (partially) working from home.
- Individual and organizational health literacy might play a crucial role in mediating the effect of organizational culture aspects on employees’ psychological wellbeing.
- Companies should include individual and organizational health literacy aspects in their mental risk assessment and focus on both when planning health promotion measures and/or corporate health management strategies.

## BACKGROUND

Health literacy *‘entails people’s knowledge, motivation and competences to access, understand, appraise, and apply health information in order to make judgments and take decisions in everyday life concerning healthcare, disease prevention and health promotion to maintain or improve quality of life during the life course’* (Sørensen *et al*., 2012). Low health literacy is associated with negative health effects (Berkman *et al*., 2011; Liu *et al.*, 2015; Svendsen *et al*., 2020), including an increased risk of death (Fan *et al.*, 2021). Several studies have indicated that a higher level of individual health literacy is positively associated with psychological wellbeing and negatively associated with depression (Tokuda *et al.*, 2009; Harsch *et al*., 2021; Zhong *et al.*, 2021; Lindert *et al.*, 2023). Despite growing awareness, such as through the National Action Plan Health Literacy in Germany, many countries still struggle with insufficient levels of health literacy among their populations (see, e.g. Nakayama *et al*., 2015; Sørensen *et al*., 2015; Schaeffer *et al.*, 2021). To empirically measure the health literacy of populations, a European research group developed a detailed questionnaire in 2012. This questionnaire was used as part of the European Health Literacy Survey (HLS), which surveyed a representative sample of individuals from eight European countries (HLS-EU Consortium, 2012). In Germany, representative surveys on the health literacy of the population were conducted in 2014 and 2020, known as HLS-GER 1 and HLS-GER 2, respectively. The surveys revealed that the majority of the German population faces major difficulties in dealing with health-related information. Alarmingly, health literacy in Germany decreased from 2014 to 2020 (Hurrelmann *et al.*, 2020). Additionally, the number of workdays lost due to mental illness increased by 52% over a 10-year period (DAK Gesundheit, 2024).

Health literacy involves not only individuals’ skills and abilities but also the requirements and complexities of the systems in which they live (Schaeffer *et al.*, 2020). Organizational health literacy is defined as *‘an organization-wide effort to transform organization and delivery of care and services to make it easier for people to navigate, understand, and use information and services to take care of their health’* (Farmanova *et al.*, 2018). Many guidelines and recommendations already exist regarding the aspects of organizational health literacy that define health-literate organizations in various settings. According to Brach *et al.* (Brach *et al*., 2012) a health-literate health care organization (i) ‘has leadership that makes health literacy integral to its mission, structure, and operations’, (ii) ‘integrates health literacy into planning, evaluation measures, patient safety, and quality improvement’, (iii) ‘prepares the workforce to be health literate and monitors progress’, (iv) ‘includes populations served in the design, implementation, and evaluation of health information and services’, (v) ‘meets the needs of populations with a range of health literacy skills while avoiding stigmatization’, (vi) ‘uses health literacy strategies in interpersonal communications and confirms understanding at all points of contact’, (vii) ‘provides easy access to health information and services and navigation assistance’, (viii) ‘designs and distributes print, audiovisual, and social media content that is easy to understand and act on’, (ix) ‘addresses health literacy in high-risk situations, including care transitions and communications about medicines’, and (x) ‘communicates clearly what health plans cover and what individuals will have to pay for services’. In a literature review, Bremer *et al.* (Bremer *et al*., 2021) identified six main categories of criteria that characterize a health-literate health care organization: communication with service users, easy access and navigation, integration and prioritization of organizational health literacy, assessments and organizational development, engagement and support of service users, and information and qualification of staff. To address organizational health literacy in the school setting, Kirchhoff *et al.* (Kirchhoff *et al*., 2022) developed the HeLit-Schools concept comprising eight standards: include health literacy into the school’s mission statement, health literacy as part of school development, promote and enhance health literacy in daily school life, health literacy of students, health-literate school staff, health-literate communication at school, enhance health literacy in the school environment, and networking and cooperation. For organizations in general, the Austrian Health Literacy Alliance (Österreichische Plattform Gesundheitskompetenz, Gesundheit Österreich, 2019) proposes nine sub-processes towards a health-literate organization: gaining leadership of the organization and actively shaping leadership responsibility, create supportive framework conditions and incentives for change in the organization, inform and involve employees, specialist support, nominate a health literacy officer and form a health literacy team, as-is analysis and target derivation, action planning and implementation, qualify employees, and evaluation and permanent anchoring. According to Sørensen *et al. ‘placing greater emphasis on health literacy outside of healthcare settings has the potential to impact on preventative health and reduce pressures on health systems’* (Sørensen *et al*., 2012). In line with this recommendation, the National Action Plan Health Literacy in Germany emphasizes the promotion of health literacy at work (Schaeffer *et al*., 2021). In the workplace, a high level of individual health literacy may positively impact employees’ work ability and reduce occupational hazards and risks (Hamacher *et al.*, 2012; Ehmann *et al.*, 2021; Stassen *et al*., 2021). A recent study found that organizational health literacy is positively associated with employees’ psychological wellbeing and serves as a mediator in the relationship between individual health literacy and employees’ psychological wellbeing (Lindert *et al*., 2023). Despite the emerging body of research on individual and organizational health literacy, empirical studies on organizational health literacy in workplace settings remain relatively rare (Lindert *et al.*, 2022). Additionally, there is a lack of empirical research examining the interaction between individual and organizational health literacy at work.

Since the onset of the SARS-CoV-2 pandemic, the number of employees working from home has increased. The majority of employees prefer not to return to office-based employment entirely (Frodermann *et al*., 2021). Not providing the option to work from home may negatively affect an employer’s branding and influence job seekers’ decisions regarding potential employers. As a result, many organizations continue to offer options for (partial) remote work (Weber *et al.*, 2022). In Germany, employees work an average of one day from home per week, which is above the global average of 0.9 days. In comparison to other European countries, Germany ranks first, along with Finland and the Netherlands (Aksoy *et al*., 2023). Working from home presents a range of complexities, as the extent and nature of work-from-home arrangements vary across different jobs and industries. Options can include working from home one day a week or doing so full-time. Yang *et al.* differentiate between ‘extension work from home’, which refers to working beyond regular hours, and ‘replacement work from home’, which occurs during regular hours (Yang *et al.*, 2023). Their research shows that while extension work from home is negatively associated with psychological wellbeing, replacement work from home positively affects both psychological wellbeing and job satisfaction. In addition to these positive effects—such as a reduced risk of work–family conflicts, improved employee performance, and increased job and life satisfaction (Beermann *et al.*, 2019)—working from home also poses new challenges for both employers and employees. For instance, it may lead to reduced social interaction (Beermann *et al*., 2019; Neumann *et al.*, 2021; Seinsche *et al.*, 2021), which is vital for maintaining mental health at work (Rothe *et al*., 2017). Moreover, employees might struggle with a lack of recovery if they work at atypical hours that encroach on their downtime (Hofmann *et al.* 2019). The blurring of boundaries between work and personal life—evidenced by a lack of physical separation and the feeling of being ‘constantly available’ (Neumann *et al*., 2021; Seinsche *et al*., 2021)—can lead to increased psychological stress. This scenario demands a heightened level of self-management and time management from employees, necessitating the development of a personal work schedule and adherence to designated working hours (Rastetter, 2020). Furthermore, employees may encounter communication challenges with colleagues and supervisors, along with difficulties in the accessibility and coordination of processes. Issues such as a lack of trust from managers or colleagues, a negative perception of remote work, and a prevalent ‘on-site culture’ (where long hours at the office are viewed as a sign of loyalty and commitment) can exacerbate these challenges (Rastetter, 2020; Neumann *et al*., 2021; Seinsche *et al*., 2021). In this context, cultivating a positive ‘work-from-home culture’—characterized by strong trust in employees, respectful communication, and effective organization of targets and regulations regarding availability and work tasks—can serve as a valuable resource for employees’ mental health. Such a culture has been found to be positively associated with psychological wellbeing and negatively associated with emotional exhaustion (Seinsche *et al*., 2021).

In Germany, mental illnesses are the second most common reason for work absences (Knieps *et al.*, 2020). According to the Occupational Health and Safety Act, employers in Germany are obliged to identify and prevent or mitigate possible risks to their employees’ mental health. They must also provide support through occupational integration management for employees who are absent due to illness for 6 weeks or more within a year (Wanek and Schreiner-Kürten, 2021). However, with demographic changes and a shortage of skilled workers, maintaining employees’ psychological wellbeing is important not only for legal compliance (Sauer and Wollmershäuser, 2021). The rise in work from home presents challenges for employers, as health and safety measures initially designed for the workplace may be less effective or fail for employees working from home. This situation necessitates a reevaluation and adaption of occupational health and safety measures (Eickholt *et al.*, 2015; Winter and Seitz, 2017). Current research suggests that the overall concept of health literacy is a strong foundation for enhancing employees’ psychological wellbeing, as both individual and organizational health literacy have been positively associated with employees’ psychological wellbeing (Lindert *et al*., 2023). Additionally, both individual and organizational perspectives play a significant role in influencing employees’ psychological wellbeing (e.g. Rothe *et al*., 2017; Hamilton Skurak *et al.*, 2021; Hati and Pradhan, 2021).

This study is the first to explore the relationships between individual and organizational health literacy, work-from-home culture, and the psychological wellbeing of employees who primarily (>50%) work from home in Germany. Based on current research, we hypothesize that individual and organizational health literacy and work-from-home culture are positively associated with employees’ psychological wellbeing. Additionally, we propose that organizational health literacy acts as a mediator in the relationship between individual health literacy and employees’ psychological wellbeing. Furthermore, we examined how individual and organizational health literacy influence the relationship between work-from-home culture and employees’ psychological wellbeing.

## METHODS

In June 2023, an anonymous open online survey was conducted using the web-based online survey tool LimeSurvey. Facebook and Instagram advertisements (see Supplementary Appendix) informed potential participants about the survey, and directed them to the survey landing page via a clickable link. Facebook’s algorithms targeted users of all genders, aged 18 and older, who were identified as employees in Germany, ensuring the advertisements reached the appropriate audience. The survey was voluntary and aimed at employees in Germany who work from home more than 50% of their working hours. Participants were informed about the length of the survey (approx. 15 min), the study purpose, the research team (including contact details) and data storage practices. Participants had to confirm their consent by ticking a provided checkbox before the actual survey began. The first two questions assessed eligibility, requiring respondents to confirm that they worked more than 50% of their time from home and were currently employed. Those who indicated they worked less than 50% from home or were exclusively self-employed were thanked for their interest and informed that they were not eligible to participate. Prior to launching the questionnaire, both the web-based and mobile versions were tested on usability and technical functionality. Adaptive questioning was used to reduce the item number and survey complexity. Items were not randomized or alternated. Participants were made aware that completing all questions was not mandatory to finish the survey. No incentives were provided for participation. The online questionnaire was divided into nine thematic groups, showing one group per site with three to a maximum of 16 items. The approval for the study was waived by the Ethics Committee of the Brandenburg Medical School.

## STUDY SAMPLE

In total, 99 000 individuals saw the advertisement on Facebook or Instagram. Of those, 232 participated in the survey (recruitment rate = 0.23%). A total of 103 employees answered all main relevant variables (psychological wellbeing, organizational health literacy, individual health literacy and work-from-home culture) (completeness rate = 44.4%). Of those, 17 were males and 86 females. The employees were employed in various working sectors, including the health sector (17.5%), business-related services (14.6%), IT and natural science services (13.6%), and social and cultural services, and education (12.6%). Most of them were working in large companies with more than 500 employees (59.2%), whereas 13.6% worked in small companies with less than 50 employees. Employees were more likely to be in full-time employment (67%) and 14.6% of participants held a management position (see Table 1). On average, participants worked 80.8% of their working hours from home. The mean age was 49.5 years (SD = 9.73).

**Table 1:** Descriptive of study sample grouped by percentage of total working hours worked from home

	Percentage of total working hours worked from home^*^								Total per category
	55–75%				80–100%				
	*N* (in %)				*N* (in %)				*N* (in %)
Company size									
<50 employees	7 (50%)				7 (50%)				14 (14%)
50 to 99 employees	2 (29%)				5 (71%)				7 (7%)
100 to 249 employees	3 (75%)				1 (25%)				4 (4%)
250 to 500 employees	7 (58%)				5 (42%)				12 (12%)
* *More than 500 employees	22 (36%)				39 (64%)				61 (62%)
Employment situation									
Full-time	26 (38%)				43 (62%)				69 (67%)
Part-time	16 (50%)				16 (50%)				32 (31%)
Marginal part-time (‘mini job’)	2 (100%)				0 (0%)				2 (2%)
Leadership position									
Yes	8 (53%)				7 (47%)				15 (15%)
No	36 (41%)				52 (59%)				88 (85%)
Gender									
Male	5 (29%)				12 (71%)				17 (17%)
Female	39 (45%)				47 (55%)				86 (83%)
Branch									
Industry and production	2 (40%)				3 (60%)				5 (5%)
Food and restaurant industry	0 (0%)				3 (100%)				3 (3%)
Health sector	9 (50%)				9 (50%)				18 (17%)
Social and cultural services and education	5 (38%)				8 (62%)				13 (13%)
Trade	3 (75%)				1 (25%)				4 (4%)
Business-related services	5 (33%)				10 (67%)				15 (15%)
IT and natural science services	6 (43%)				8 (57%)				14 (14%)
Security, transport and logistics	0 (0%)				3 (100%)				3 (3%)
Other	14 (50%)				14 (50%)				28 (27%)

	*N*	*M*	SD	Median	*N*	*M*	SD	Median	*t*-test *p*-value
Age	44	50.09	9.56	51.00	59	49.05	9.91	50.00	0.594
Individual health literacy	44	3.05	0.56	3.05	59	3.07	0.42	3.00	0.869
Organizational health literacy	44	2.51	0.83	2.50	59	2.66	0.89	2.67	0.366
Work-from-home culture	44	2.85	0.66	2.90	59	3.11	0.77	3.20	0.076
Psychological wellbeing	44	11.16	6.47	12.00	59	10.00	5.56	10.00	0.332

*N* = number of observations; *M* = mean; *SD* = standard deviation.

^*^As the questionnaire slider could only be moved in 5% steps, answers between 75% and 80% were not possible.

## MEASURES

To measure the *individual health literacy* of employees, we utilized items from the Health Literacy Scale for Workers (Azizi *et al.*, 2019). These items were translated into German and adapted for the study objectives according to the TRAPD process: (i) The original items were translated into German by two independent translation agencies. (ii) The research team, consisting of LaK, LuK and KEC, reviewed the translated versions and agreed on a preliminary version. (iii) The survey was pretested by seven individuals who met the criteria of the target group. (iv) Feedback from the pretesters was discussed among LaK, LuK and KEC, leading to a consensus on a final version of the questionnaire. This process ensured that the questions were culturally appropriate and understandable within the German context. The final scale consists of 13 items that could be answered on a 4-point Likert scale (1—‘I do not agree at all’ to 4—‘I totally agree’), e.g. ‘I always take care of my health in any activities’ and ‘I can ask others (e.g. friends, doctors, colleagues) about the health information I need’. Cronbach’s alpha of the individual health literacy scale was 0.8. *Organizational health literacy* was operationalized by six items that could be answered on a 4-point Likert scale (1—‘I do not agree at all’ to 4—‘I totally agree’). The scale aligns with the definition of organization health literacy provided by Farmanova *et al.* and aims to assess employees’ perception of their companies’ efforts in providing information, as well as in supporting and involving employees in health promotion initiatives (Farmanova *et al*., 2018). The scale was initially developed for an employee survey at a large German financial institution. For further details, refer to Lindert *et al.* (Lindert *et al*., 2023). Cronbach’s alpha was 0.9. *Employees’ psychological wellbeing* was assessed using the WHO-5 Well-Being Index (World Health Organization, 1998) that comprises five items rated on a 6-point scale ranging from 0 (‘never’) to 5 (‘the whole time’). The items are designed to capture a person’s feelings over the past 2 weeks, e.g. ‘Over the last two weeks I have felt cheerful and in good spirits’. The raw score can range from 0 to 25. A score below 13 is considered an indicator that further testing for depression may be warranted (Löwe *et al*., 2004). Cronbach’s alpha was 0.9. *Work-from-home culture* encompasses the strategies and regulations for working from home, supervisor training and support for employees working from home, and promotion of and information about the possibility of working from home (Neumann *et al*., 2021; Seinsche *et al*., 2021). The scale includes five items that could be answered on a 4-point Likert scale (1—‘I do not agree at all’ to 4—‘I totally agree’), e.g. ‘Our organization has strategies and regulations for working from home’. Cronbach’s alpha was 0.8. As only employees who worked more than 50% from home could participate in this study, the *percentage of total working hours worked from home* could be answered on a slide scale ranging from 55% to 100% (the slider could be moved in 5% steps).

## DATA ANALYSIS

The aim of the analyses was three-fold: (i) to examine the relationship between individual health literacy, organizational health literacy and work-from-home culture with employees’ psychological wellbeing, (ii) to investigate whether organizational health literacy mediates the effect of individual health literacy on employees’ psychological wellbeing and (iii) to assess how both individual and organizational health literacy influence the relationship between work-from-home culture and employees’ psychological wellbeing. Descriptive and inferential statistical data analyses were conducted using IBM SPSS Statistics 23. Descriptive analyses, including mean values, standard deviations and frequencies, were employed to evaluate participants’ level of individual and organizational health literacy, psychological wellbeing, work-from-home culture and other relevant variables such as age and gender. Simple and parallel mediation analyses were performed using the PROCESS Macro by Hayes (Hayes, 2022; v4.1). PROCESS uses ordinary least squares regressions, yielding unstandardized path coefficients for total, direct and indirect effects. Bootstrap inference for model coefficients was computed with 5000 bootstrap samples to compute confidence intervals and inferential statistics, and heteroscedasticity-consistent inference was calculated according to Davidson and MacKinnon (Davidson and MacKinnon, 1993). The significance of indirect effects was assessed according to Sobel (Sobel, 1982), and effects were considered significant when the confidence interval did not include zero.

## RESULTS

Table 1 presents the descriptive statistics of the study sample, categorized by the percentage of total working hours spent working from home. For the continuous variables—age, individual and organizational health literacy, work-from-home culture and psychological wellbeing—group differences were analyzed using *t*-tests. The tests showed no significant differences between employees working 55% to 75% from home and those working 80% to 100% from home in terms of age (*t*(101) = 0.594, *p* > 0.05), individual health literacy (*t*(101) = −0.166, *p* > 0.05), organizational health literacy (*t*(101) = −0.908, *p* > 0.05), work-from-home culture (*t*(101) = −1.794, *p* > 0.05) and psychological wellbeing (*t*(101) = 0.976, *p* > 0.05). Due to small subgroup sizes, chi-square tests could not be applied for the ordinal variables—company size, employment situation, leadership position, gender and branch. Additionally, the underlying data did not indicate any noticeable trends for these variables (see Table 1).

**Fig. 1.**
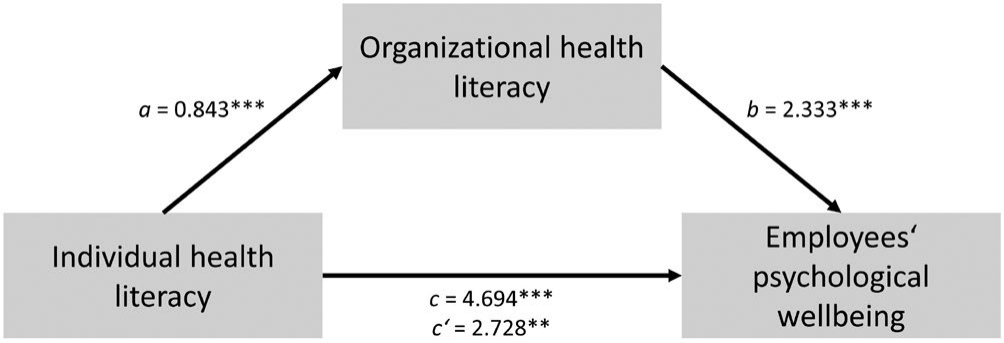
Mediation model 1.

In the total sample, the mean scores were as follows: individual health literacy had a mean of 3.1, organizational health literacy had a mean of 2.6, work-from-home culture had a mean of 3.0, and psychological wellbeing had a mean of 10.5. In mediation model 1, a significant total effect of individual health literacy on employees’ psychological wellbeing was found, *c* = 4.694, *p* < 0.001. Additionally, individual health literacy significantly predicted organizational health literacy, *a* = 0.843, *p* < 0.001. Meanwhile, organizational health literacy significantly predicted employees’ psychological wellbeing, *b* = 2.333, *p* < 0.001 (see Figure 1 and Table 2). The relationship between individual health literacy and employees’ psychological wellbeing was found to be partially mediated by organizational health literacy, indirect effect *ab* = 1.967, *p* < 0.001 (see Table 2).

**Table 2:** Coefficient of effect, Se, LLCI, ULCI for mediation models 1 and 2

Dependent variable = PW	Coefficient of effect	SE	LLCI	ULCI
*Model 1*				
* *Direct effect of IHL on mediator OHL (*a*)	0.843^***^	0.154	0.536	1.148
* *Direct effect of IHL on PW (*c’*)	2.728^**^	0.979	0.785	4.671
* *Direct effect of OHL on PW (*b*)	2.333^***^	0.648	1.047	3.619
* *Indirect effect of IHL on PW (*ab*)	1.967^***^	0.655	0.773	3.396
* *Total effect on PW (*c*)	4.694^***^	1.024	2.664	6.725
*Model 2*				
* *Direct effect of WHC on mediator OHL (*a*^1^)	0.731^***^	0.088	0.557	0.905
* *Direct effect of WHC on mediator IHL (*a*^2^)	0.249^**^	0.081	0.089	0.410
* *Direct effect of WHC on PW (*c’*)	0.653	0.836	−1.006	2.312
* *Direct effect of OHL on PW (*b*^1^)	2.018^**^	0.738	0.554	3.483
* *Direct effect of IHL on PW (*b*^2^)	2.624^*^	1.002	0.635	4.614
* *Indirect effect of WHC on PW (*a*^1^*b*^1^)	0.654^**^	0.334	0.089	1.388
* *Indirect effect of WHC on PW (*a*^2^*b*^2^)	1.475^***^	0.558	0.366	2.593
* *Total effect on PW (*c*)	2.783^***^	0.740	1.314	4.251

SE = standard error, LCI = lower level confidence interval, ULCI = upper level confidence interval, IHL = individual health literacy, OHL = organizational health literacy, PW = psychological wellbeing, WHC = work-from-home culture.

^*^ *p* < 0.05,

^**^ *p* < 0.01,

^***^ *p* < 0.001.

**Fig. 2.**
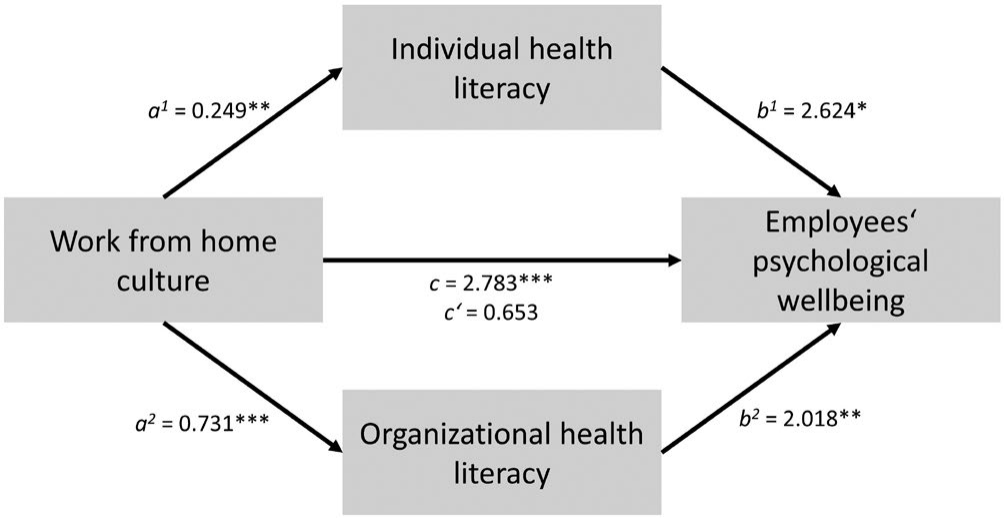
Mediation model 2.

In mediation model 2, a significant total effect of work-from-home culture on employees’ psychological wellbeing was found, *c* = 2.783, *p* < 0.001. Work-from-home culture significantly predicted individual health literacy, *a*^1^ = 0.249, *p* < 0.01, and organizational health literacy, *a*^2^ = 0.731, *p* < 0.001. Both individual and organizational health literacy were significant predictors of employees’ psychological wellbeing, *b*^1^ = 2.624, *p* < 0.05, *b*^2^ = 2.018, *p* < 0.01 (see Figure 2 and Table 2). The relationship between work-from-home culture and employees’ psychological wellbeing was totally mediated by individual and organizational health literacy, indirect effect *a*^1^*b*^1^ = 0.654, *p* < 0.01 and *a*^2^*b*^2^ = 1.475, *p *< 0.001, indicating an indirect-only mediation (see Table 2).

## DISCUSSION

In line with previous studies (Tokuda *et al*., 2009; Harsch *et al*., 2021; Neumann *et al*., 2021; Seinsche *et al*., 2021; Zhong *et al*., 2021; Lindert *et al*., 2023), our findings revealed that individual and organizational health literacy and work-from-home culture are positively associated with employees’ psychological wellbeing. Furthermore, organizational health literacy mediates the effect of individual health literacy on employees’ psychological wellbeing. Additionally, we examined how individual and organizational health literacy affect the relationship between work-from-home culture and employees’ psychological wellbeing. In our study sample, the impact of work-from-home culture was totally mediated by individual and organizational health literacy. While organizational health literacy has primarily been studied in health care settings (Lindert *et al*., 2022), our results suggest that both individual and organizational health literacy are valuable concepts for practitioners and researchers focused on promoting the psychological wellbeing of employees working from home.

The findings are not only relevant for Germany but also for other countries. The results of the HLS-EU, for instance, indicate that many countries report low levels of health literacy among their populations. Furthermore, similar legal requirements regarding health protection at work exist in other nations. For instance, mental risk assessments are mandatory in Belgium, Bulgaria, Italy, Latvia, Lithuania, the Netherlands, Portugal, Hungary, and Cyprus. Working from home is also prevalent in various countries. While Germany has one of the highest numbers of home office days in Europe, English-speaking countries (such as Australia, Canada, the USA and the UK) lead globally (Aksoy *et al*., 2023).

Key factors for the successful organization-wide implementation of organizational health literacy interventions include leadership support, both top-down and bottom-up approaches, the presence of a change champion and staff commitment (Kaper *et al.*, 2021). Essential mechanisms that facilitate implementation are staff knowledge of organizational health literacy, internal expertise in health literacy, shared responsibility and a systematic implementation strategy (Meggetto *et al.*, 2020). According to Charoghchian Khorasani *et al.* (Charoghchian Khorasani *et al.,* 2020), barriers to effective implementation of organizational health literacy interventions in healthcare centers include: poor organizational commitment to health literacy; time and resource constraints; lack of policies, procedures and protocols supporting health literacy practices; overemphasis on patient engagement and self-management without broader organizational health literacy focus; health environments that lack health literacy-friendly features; poor interactive and linguistic skills, including the use of overly technical language, abbreviations or inconsistent terminology; ambiguity in roles among healthcare providers and staff in addressing health needs and materials; deficient verbal and written communication skills; patients’ lack of confidence in completing medical forms; resistance to a culture of change and innovation; limited patient involvement in health literacy efforts; lack of staff training or knowledge about health literacy and related activities; poor navigation systems within healthcare facilities (e.g. unclear signage, confusing layouts); financial constraints. Eickholt *et al.* (Eickholt *et al*., 2015) highlight the importance of organizational development, empowerment and the setting approach—where settings (co-)determine individual behavior—for enhancing health literacy in companies. Hauer *et al.* (Hauer *et al.,* 2018) emphasize the role of company networks. By fostering networks within workplace health promotion, companies can amplify learning and dissemination effects, thereby increasing organizational health literacy. This occurs as training participants share their knowledge within their organizations, contributing to a greater overall understanding of health literacy in the workplace. As work from home may lead to ‘technostress’ due to the challenges associated with digitalization and mobile forms of work, Begerow *et al*. emphasize the importance of organizing work effectively with respect to technology (Begerow *et al.*, 2020). They suggest that both employees and managers should receive support in developing health literacy to deal with (new) ICT and the associated forms of work in a healthy manner. Additionally, they assert that successfully organizing working from home necessitates attention to several key areas: organization of work, working tasks, social relationships at work, work environment and organizational culture. Our study results indicate that it may be beneficial to also prioritize individual and organization health literacy, as these factors could significantly mediate the relationship between aspects of organizational culture and employees’ psychological wellbeing.

In this study, no association was found between the amount of time employee’s work from home and their psychological wellbeing. These findings may be supported by the research of van der Lippe *et al.*, which suggests that the relationship between work from home time and psychological wellbeing is curvilinear (van der Lippe *et al.*, 2019). Specifically, wellbeing tends to be lower among employees who either rarely or often work from home. Additionally, no significant results were observed regarding the link between work from home time and individual and organizational health literacy, indicating that health literacy may not be relevant in relation to employees’ work from home time. Regarding the association between work from home time and work-from-home culture, there was a slight tendency observed—although not significant—indicating that a better work-from-home culture may correlate with increased work from home time. However, these findings warrant further investigation in future studies, as underlying effects might be small and the study sample is not large enough to reveal associations.

The psychological wellbeing in our study sample, with a mean of 10.5, is relatively low compared to employees in other studies (e.g. Zeike *et al.*, 2018; Russo and Terraneo, 2020; Lindert *et al.*, 2022, Lindert *et al.*, 2022). This may be attributed to the fact that the majority of the participants in our study identified as female. Several studies have demonstrated that women tend to report lower psychological wellbeing compared to men (Sio *et al*., 2017; Gómez-Baya *et al.*, 2018; Etheridge and Spantig, 2020). However, even compared with studies that examined gender differences using the WHO-5 Well-Being Index (Sio *et al*., 2017; Gómez-Baya *et al*., 2018), the psychological wellbeing of our study sample still appears low. Additionally, Lee found that men reported lower psychological wellbeing than women (Lee, 2015). These findings suggest that the low psychological wellbeing in our study sample may not be solely attributed to gender aspects but could be influenced by other underlying mechanisms. For instance, it might support the conclusions of van der Lippe et al., indicating that the psychological wellbeing of our participants could be lower due to a significant number of them working from home often (>80%) (van der Lippe *et al*., 2019). However, this needs to be investigated in future research.

## STRENGTHS AND LIMITATIONS

This study is, to our knowledge, the first to investigate individual and organizational health literacy among employees primarily working from home. However, it has certain limitations that prevent the results from being representative or generalizable for all employees. The small sample size may have hindered the detection of statistically significant results, such as differences in age, individual health literacy, organizational health literacy, work-from-home culture and psychological wellbeing between employees working 55% to 75% from home and those working 80% to 100% from home. Research indicates that approximately 100 cases are needed to detect medium effects and around 50 cases for large effects when assessing complete mediation with two simple mediation effects (Sim *et al.*, 2022). In this study, these thresholds were met, allowing for the observation of significant results. Additional limitations arise from the fact that most participants were female and recruitment occurred through Facebook. Although Facebook’s user base is roughly equally distributed across genders (Teschauer, 2023), the higher participation rate of female employees may be due to greater interest in the study’s topic or the advertisement’s appeal. It is also possible that women are generally more inclined to participate in online surveys than men (Smith, 2008). By using Facebook for recruitment, there is a chance that participants were more tech-savvy and familiar with technology and digital applications compared to the general employee population. Despite these limitations affecting representativeness and generalizability, study results offer valuable insights for future research in several respects.

## CONCLUSION

Since the results of this study are not generalizable, future research should investigate associations between the psychological wellbeing of employees, work-from-home culture, individual and organizational health literacy, and work from home time, using larger sample sizes. Nonetheless, the findings suggest that both individual and organizational health literacy are valuable concepts for practitioners and researchers concerning the psychological wellbeing of employees who are working from home, whether partially or fully. Both individual and organizational health literacy were positively associated with employees’ psychological wellbeing and totally mediated the impact of work-from-home culture on employees’ psychological wellbeing. Therefore, companies may benefit from incorporating elements of individual and organizational health literacy into their legally required mental risk assessments. Additionally, employers should prioritize these aspects when planning health promotion initiatives and corporate health management strategies.

## Supplementary Material

daae202_suppl_Supplementary_Appendix

## Data Availability

The data underlying this article cannot be shared publicly due to the privacy of individuals who participated in the study. The data will be shared on reasonable request to the corresponding author.
