# Supplementary figures and images for: Health literacy and psychological wellbeing of employees working from home in Germany—online survey results
Source: Health Promot Int. 2025 Jan 17;40(1):daae202. doi: 10.1093/heapro/daae202 (PMC11739716; doi:10.1093/heapro/daae202)

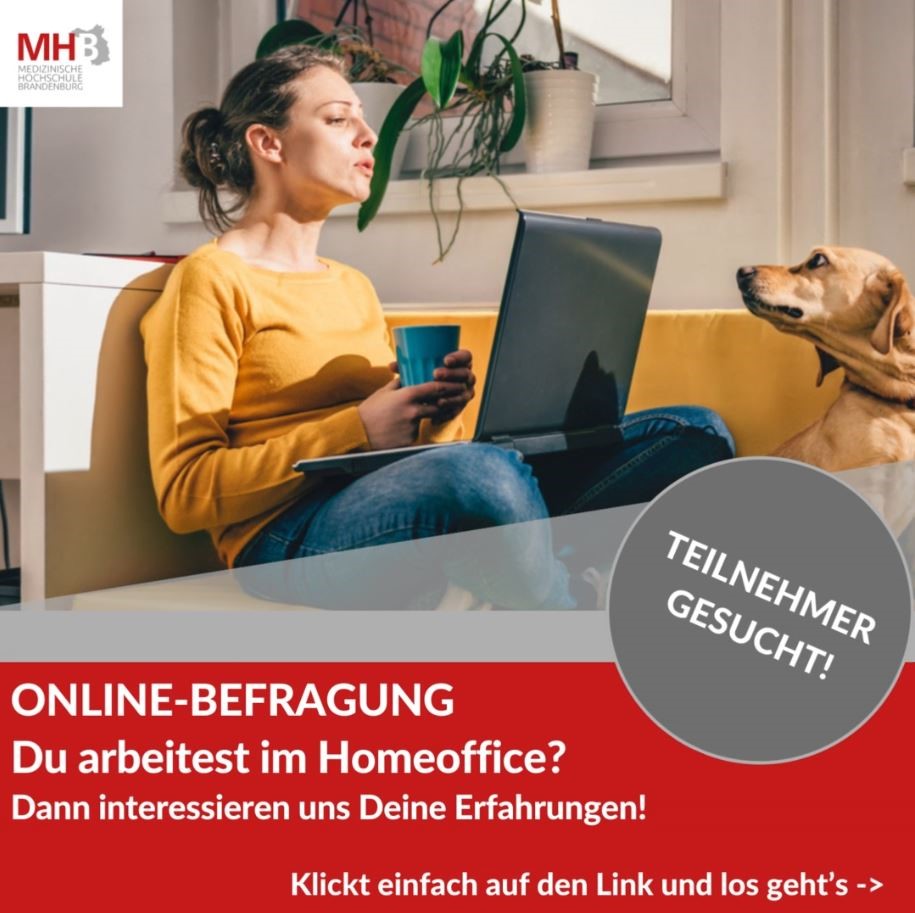

Supplement: daae202_suppl_Supplementary_Appendix [file daae202_suppl_supplementary_appendix.jpeg]
